# Surgical treatment of movement disorders in neurometabolic conditions

**DOI:** 10.3389/fneur.2023.1205339

**Published:** 2023-06-02

**Authors:** Alonso Zea Vera, Andrea L. Gropman

**Affiliations:** ^1^Division of Neurology, Children’s National Hospital, Washington, DC, United States; ^2^Department of Neurology, George Washington University School of Medicine and Health Sciences, Washington, DC, United States; ^3^Division of Neurogenetics and Neurodevelopmental Pediatrics, Children’s National Hospital, Washington DC, United States

**Keywords:** movement disorder, dystonia, neurometabolic disorder, deep brain stimulation, pallidotomy, thalamotomy

## Abstract

Refractory movement disorders are a common feature of inborn errors of metabolism (IEMs), significantly impacting quality of life and potentially leading to life-threatening complications such as status dystonicus. Surgical techniques, including deep brain stimulation (DBS) and lesioning techniques, represent an additional treatment option. However, the application and benefits of these procedures in neurometabolic conditions is not well understood. This results in challenges selecting surgical candidates and counseling patients preoperatively. In this review, we explore the literature of surgical techniques for the treatment of movement disorders in IEMs. Globus pallidus internus DBS has emerged as a beneficial treatment option for dystonia in Panthotate-Kinase-associated Neurodegeneration. Additionally, several patients with Lesch–Nyhan Disease have shown improvement following pallidal stimulation, with more robust effects on self-injurious behavior than dystonia. Although there are numerous reports describing benefits of DBS for movement disorders in other IEMs, the sample sizes have generally been small, limiting meaningful conclusions. Currently, DBS is preferred to lesioning techniques. However, successful use of pallidotomy and thalamotomy in neurometabolic conditions has been reported and may have a role in selected patients. Surgical techniques have also been used successfully in patients with IEMs to treat status dystonicus. Advancing our knowledge of these treatment options could significantly improve the care for patients with neurometabolic conditions.

## 1. Introduction

Inborn errors of metabolism (IEMs) are a group of genetic disorders caused by deficiencies in a metabolic pathway, leading to the accumulation of toxic substrates or depletion of essential compounds. These conditions are characterized by multisystemic symptoms, with a high prevalence of central nervous system involvement (1, 2). Movement disorders are a common feature of IEMs, with dystonia being a frequently observed manifestation (2, 3). Dystonia is characterized by sustained or intermittent muscle contractions causing abnormal movements, postures, or both. These movements are typically patterned and twisting, and occasionally tremulous (4). Despite the emphasis on early diagnosis and prevention of neurologic injury, many IEMs do not have a specific treatment or patients are not identified until irreversible damage has occurred (3, 5). A significant proportion of these patients experience severe and pharmacologically resistant movement disorders that significantly impair their quality of life (QoL). Status dystonicus, a life-threating complication, can also present in this population (6, 7). As a result, effective treatments for movement disorders associated with IEMs represent a pressing clinical need.

Surgical interventions play a critical role in the management of refractory movement disorders. Techniques such as deep brain stimulation (DBS) and lesioning procedures, including pallidotomy and thalamotomy, have demonstrated considerable clinical benefit (8, 9). Currently, DBS has largely replaced lesioning techniques. Randomized controlled trials have shown DBS to be effective in treating tremor, Parkinson’s disease, and dystonia (10–12). Observational studies have also reported promising results of DBS in patients with tics, myoclonus, and chorea (13–15). Although less frequently than in Parkinson’s disease, DBS has also been used for other synucleopathies (16). Moreover, DBS has shown a positive effect in the treatment of some psychiatric conditions including self-injurious behaviors (SIB) and obsessive–compulsive disorder (OCD) (17, 18). Traditionally, DBS has been indicated based mainly on phenotype. However, with the advancement of genetic testing, it has become evident that genotype can play a significant role in predicting the effect of DBS. Consequently, studies have demonstrated differential effects of DBS in monogenetic dystonias and Parkinson’s disease, highlighting the importance of considering genetic factors in treatment outcomes (19, 20).

Deep brain stimulation presents a potential treatment option for patients with IEMs who commonly present with dystonia, other movement disorders, and comorbid psychiatric conditions. However, several factors have limited its use in this population. First, structural brain lesions, often located in the basal ganglia, are frequently observed in patients with IEMs. The efficacy of DBS for dystonia associated with structural lesions is more variable and less robust compared to so-called primary dystonia, leading to hesitation in patients and providers (19). Second, DBS does not affect the progression of the underlying disease. Therefore, a gradual loss of effect over time is expected due to the progressive nature of many of these conditions, questioning the long-term value of the intervention. Finally, anesthesia and surgery may trigger metabolic crises in susceptible patients, thus increasing the risk of DBS. Despite these limitations, an increased understanding of the indications, safety, and efficacy of surgical therapies for movement disorders in IEMs would be beneficial to improve QoL for this complex patient population.

In this review, we explore the existing literature on surgical interventions for movement disorders in IEMs, with a focus on DBS as the more prevalent technique. However, we also briefly discuss reports of lesioning procedures in this population. A PubMed search was performed using combinations of the following terms: “Deep Brain Stimulation”[Mesh], “Pallidotomy”[Mesh], “Neurosurgical Procedures”[Mesh], Deep Brain Stim*, Pallidotomy, Thalamotomy, “Metabolism, Inborn Errors”[Mesh], Neurometabol*, Inborn Errors of Metabolism, Neurodegeneration with brain iron accumulation, Lesch–Nyhan Disease, Mitochondrial, Pantothenate kinase-associated neurodegeneration, Wilson Disease. The references of selected articles were reviewed to identify additional relevant studies.

## 2. Neurodegeneration with brain iron accumulation

Neurodegeneration with Brain Iron Accumulation (NBIA) is a heterogenous group of inherited disorders characterized by anomalous iron deposition in the basal ganglia (21). The most prevalent form of NBIA is pantothenate kinase-associated neurodegeneration (PKAN), which results from biallelic variants in the *PANK2* gene, encoding a crucial enzyme for coenzyme A synthesis (22). PKAN manifests as progressive dystonia, spasticity, and cognitive decline. It can be divided into classic PKAN, with onset in childhood and rapid progression, and atypical PKAN, with onset in adolescence or adulthood and a more gradual course. The characteristic eye-of-the-tiger sign is frequently observed on axial T2-weighted brain MRI scans (22). Two clinical trials of disease-specific therapies did not demonstrate significant clinical benefit relative to placebo (23, 24). Thus, current management focuses primarily on symptomatic treatment (25).

Globus pallidus internus (GPi) DBS has emerged as a promising therapeutic option for dystonia in PKAN (26, 27). A recent meta-analysis encompassing 99 patients reported an average 26% improvement in dystonia severity, as measured by the Burke-Fahn-Marsden Dystonia Rating Scale (BFMDRS) motor component (BFMDRS-M), at 12 months post-operatively (26). Notably, 30% of patients experienced a decrease in dystonia severity exceeding 50%, whereas 30% experienced an improvement of less than 10% (26–28). Patients with atypical PKAN tend to have a more favorable response to DBS than those with typical PKAN (26). Despite an observed dystonia progression 2–3 years post-surgery, many patients followed for up to 5 years still exhibited lower BFMDRS-M scores relative to pre-operative assessments (29–33). DBS has also been efficacious in treating pharmacologic-resistant status dystonicus in emergency scenarios (6, 34, 35).

The impact of DBS on the disability component of the BFMDRS (BFMDRS-D) is more limited, with no significant mean benefit in typical PKAN and a moderate effect in atypical PKAN (26). However, the BFMDRS-D has limitations in capturing critical aspects that adversely affect QoL, such as pain. Studies evaluating QoL have reported improvements of up to 80% 1 year post-surgery, with the greatest impact on pain and mobility (27, 36). Interestingly, improvements in cognitive tests after surgery have also been reported. This observation suggests that the cognitive decline observed in PKAN patients may be due in part to the challenges in accessing and completing cognitive tests caused by the severity of dystonia (33, 37, 38).

Alternative DBS targets may present a promising option for dystonia treatment in PKAN, particularly considering the frequent occurrence of pallidal iron accumulation (39). Although limited by a small sample size, reports have shown subthalamic nucleus (STN) DBS to result in BFMDRS-M improvements ranging from 46 to 87% at the 12-month postoperative mark (40–42). The posterior ventrolateral thalamus has also been infrequently utilized to treat PKAN dystonia (43). Outside PKAN, single cases of successful use of DBS for dystonia in mitochondrial membrane protein-associated degeneration, *PLA2G6*-associated neurodegeneration, and Woodhouse-Sakati syndrome have been reported (44–46). Furthermore, DBS has also been used to address severe OCD, bradykinesia, and tremor in NBIA patients with negative genetic testing (47, 48).

## 3. Lesch–Nyhan disease

Lesch–Nyhan disease (LND) is caused by loss of the purine salvage enzyme hypoxanthine-guanine phosphoribosyltransferase (*HPRT*), leading to hyperuricemia, dystonia, spasticity, cognitive impairment, and SIB. The efficacy of GPi-DBS in LND has been studied in 12 patients with a follow-up period ranging from 6 months to 12 years, with a median of 2 years and 5 months (49–57). In 9 of these patients pre- and post-operative BFMDRS-M scores were available, with a mean improvement of 20%. One patient improved by > 50% and 4 patients showed less than 10% improvement (51, 56, 58). The remaining 3 patients without BFMDRS reported a significant subjective improvement of dystonia (50, 52). Remarkably, all patients experienced an improvement in SIB, with complete resolution in some cases (55, 56, 58). Adverse events were reported in half the patients (57).

The therapeutic effect of DBS on SIB is influenced by the neuroanatomical location of stimulation. One patient with bilateral GPi-DBS that suffered a unilateral lead fracture had a recurrence of SIB limited to the contralateral side of the body. In another patient with bilateral anterior and posterior GPi electrodes, it was noted that SIB, but not dystonia, recurred when only the anterior stimulation was turned off (53, 54). Despite the posterior GPi being the primary target for dystonia, the anterior GPi, which has stronger limbic connections, may play a more critical role in managing SIB. Consequently, dual GPi stimulation has been suggested to be more effective for managing both dystonia and SIB. However, due to the lack of empirical evidence showing advantages over single GPi stimulation and the increased risk of adverse events associated with additional leads, it has not been widely adopted (51, 52, 54, 57).

While the efficacy of DBS in LND seems promising, there are indications that publication bias may be inflating the benefits. A survey of caregivers of 14 LND patients that received DBS, recruited from different patient groups, revealed more modest benefits (58). The mean time since surgery was 5.5 years and 6 patients had discontinued DBS. The responses leaned slightly towards improvement in abnormal movement, self-injurious behaviors, oppositional behavior, apathy, and agitation, but with wide variability (58). Thirteen patients reported adverse effects from DBS (58). Additionally, a recent large cohort of French and Italian *HPRT*-deficient patients briefly mentioned four patients with disappointing DBS results, but no specific information on surgery or outcomes was provided (59). Due to the limited information, these patients are not generally included in systematic reviews (57, 58).

## 4. Mitochondrial diseases

Mitochondrial diseases are a group of conditions that are genetically and clinically diverse, often resulting in complex movement disorders that are difficult to treat. Bilateral GPi-DBS has been reported as an effective management strategy for dystonia in patients with variants in *SLCA25A42*, *TWNK*, *TIMM8A*, and multiple mitochondrial DNA deletions (60–63). When reported, improvements in the BFMDRS-M ranged from 36 to 64%, with a follow-up of 1–2 years. Two cases with *TWNK* mutation also noted mild improvement of parkinsonian symptoms, one of which also had bilateral STN-DBS (61). It is worth noting that none of the patients treated with GPi-DBS had pallidal lesions on brain imaging. Additionally, DBS with a thalamic target has been employed to manage severe postural-kinetic tremor in a patient with biopsy-confirmed mitochondrial disease after multiple stroke-like episodes (64).

Despite reports showing benefits from DBS, there have also been cases with disappointing outcomes. For instance, in one patient with biopsy-confirmed complex I deficiency presenting with dystonia and myoclonus, an initial reduction in myoclonus was observed, but symptoms recurred by 12 months after bilateral GPi-DBS. Only minimal effect on dystonia was noted (65). Another patient with multiple mitochondrial deletions presenting with rapidly progressive dystonia-parkinsonism also had only a brief and temporary response after bilateral GPi-DBS. Additional nucleus ventralis oralis anterior stimulation was attempted but also failed to have a sustained response (66).

## 5. Wilson disease

Wilson Disease (WD) is a recessive genetic disorder caused by pathogenic variants of *ATP7B* that leads to copper accumulation, resulting in hepatic, neurologic, and psychiatric problems when left untreated. Movement disorders, such as tremor, dystonia, chorea, and parkinsonism, are common among patients (67). Treatment is focused on timely diagnosis and initiation of chelation therapy to prevent permanent neurologic and hepatic injury. Unfortunately, persistent refractory movement disorders are not uncommon among patients with WD (67, 68).

Reports of DBS in WD are scarce. GPi-DBS for dystonia has shown limited benefits in a few patients, with improvements of up to 14% in the BFMDRS-M, while several patients had no response (45, 69–71). Despite these disappointing results, one report showed a much higher impact of DBS in improving caregiver burden, reflecting the limitations of the BFMDRS (69). DBS have also been used for treating tremor in WD by targeting the ventral intermediate thalamic nucleus (Vim). Reports of Vim-DBS with objective outcomes are rare and most evidence has been extrapolated from other secondary tremor literature (68). One patient who had both tremor and dystonia experienced a reduction in the Fahn-Tolosa-Marin tremor score of 97% and in the BFMDRS-M of 65% 2 years after bilateral posterior subthalamic area stimulation (PSA) (72). The PSA comprises the caudal zona incerta and the prelemniscal radiation, both of which have been found to be effective targets for tremor and bradykinesia control. Stimulation of the prelemniscal radiation has also shown promise in treating dystonia. Considering the high incidence of dystonia and tremor and associated lenticular injury in WD, targeting the PSA is an intriguing option for this patient population (72–74).

## 6. Glutaric aciduria type I

Glutaric aciduria type I (GA1) is caused by biallelic mutations of glutaryl CoA dehydrogenase. Most patients are asymptomatic or present with unspecific symptoms like hypotonia, delayed motor development, and macrocephaly early in life. If left untreated, 80–90% of infants develop irreversible striatal damage with severe generalized dystonia following an acute encephalopathic crisis (75). Treatment is focused on prevention of irreversible injury with metabolic interventions. Due to the crucial role of timely intervention before metabolic decompensation, GA1 is included in the newborn screening programs of several countries (3). GPi-DBS has been reported in a handful of patients, sometimes as a palliative measure (28, 49, 76–79). Some patients showed a modest improvement with reductions of up to 18% in the BFMDRS-M (49, 76, 77). However, a similar number of patients have failed to respond to DBS (28, 78). One report suggests that improvement in pain may be higher than in dystonia scales, but pain scales are rarely reported (80).

## 7. Other neurometabolic conditions

DBS has been used rarely as a treatment option in several other IEMs, with dystonia being the most common indication. However, there have also been reports of successful management of other movement disorders such as parkinsonism (7, 45, 70, 81–99). Table 1 provides a summary of these cases. The *GBA* gene deserves additional discussion. Biallelic pathogenic variants of *GBA* cause Gaucher’s disease, a lysosomal storage disorder. Traditionally, Gaucher’s disease has been classified based on the presence (type 2 and 3) or absence (type 1) of neurologic symptoms. However, Parkinson’s disease has been reported more frequently in patients with type 1 Gaucher’s disease (100). Reported cases of DBS this population are included in Table 1. Additionally, Parkinson’s disease patients who carry a single *GBA* variant present with earlier onset of symptoms and a more aggressive disease course. Certain variants of the *GBA* gene carry a higher risk in this context (101). While STN-DBS has been associated with good motor outcomes in this population, it has been suggested that it may have negative effects on cognitive outcomes. It remains unclear whether this observation is attributed to the DBS treatment itself, the *GBA* carrier status, or a combination of both factors. For these reasons, GPi-DBS has been proposed as a potentially cognitively safer option, although the evidence supporting this target for the GBA-associated Parkinson’s disease population is still limited (101).

**Table 1:** Summary of reported patients with rare neurometabolic conditions treated with deep brain stimulation.

	First author	Diagnosis	Movement disorder	DBS target	Follow up after surgery	Outcome
1	Racki et al. (81)	Gaucher disease	Parkinson’s disease and motor fluctuation with levodopa	Bilateral STN	36 months	Improvement of UPDRS III from 30 to 7 (77% reduction) and of UPDRS IV from 18 to 1 (94% reduction)
2	Chetrit et al. (82)	Gaucher disease	Parkinson’s disease and motor fluctuation with levodopa	NR	NR	“Good response”
3	Chetrit et al. (82)	Gaucher disease (82)	Parkinson’s disease and motor fluctuation with levodopa	NR	NR	“Good response”
4	Roze et al. (83)	GM1 gangliosidosis	Generalized dystonia and parkinsonism	Bilateral GPi	12 months	Improvement of BFMDRS-M from 70 to 56 (20% reduction) and of “akinetic score” from 37–32 (14% reduction).
5	Hasegawa et al. (84)	Adult-onset neuronal ceroid lipofuscinosis	Right hemidystonia and hemibody jerking	Left GPi	15 months	“Considerable reduction in the frequency and intensity of spasms and jerks”
6	Beaulieu-Boire et al. (45)	Neuronal ceroid lipofuscinosis	Segmental (face and neck) dystonia	Bilateral GPi	12 months	Improvement of BFMDRS-M from 34.5 to 24.5 (29% reduction) and worsening of BFMDRS-D from 12 to 13 (6% increase).
7	Elkay et al. (85)	Neuronal ceroid lipofuscinosis 3 (Batten disease)	Status dystonicus	Bilateral GPi, after bilateral pallidotomy.	7 months	Resolution of status dystonicus. Improvement of BFMDRS from 100 to 62 (38% reduction)
8	Dhar et al. (70)	Niemann Pick type C	Generalized dystonia	Bilateral GPi	24 months	Improvement of BFMDRS-M from 109 to 68 (38% reduction) in the first 12 months with subsequent worsening to 96 at 24 months (12% reduction compared to pre-surgery scores)
9	Watanabe et al. (86)	GM3 synthase deficiency	Choreoathetosis and parkinsonism	GPi, laterality NR.	13 years	Immediate dramatic improvement of choreoathetosis with recurrence in 1 week. Mild benefit since then until DBS was removed due to infection.
10	Wang et al. (87)	Autosomal dominant GTP cyclohydrolase 1 deficiency	Cervical dystonia	Bilateral GPi	8 months	Complete resolution of dystonia.
11	Daida et al. (88)	Autosomal dominant GTP cyclohydrolase 1 deficiency	Generalized dystonia and significant motor fluctuations with levodopa	Bilateral STN	NR	Improvement of UPDRS III from 12 to 9 (25% reduction) and of UPDRS IV from 18 to 3 (83% reduction).
12	Lobato-Polo et al. (7)	Autosomal dominant GTP cyclohydrolase 1 deficiency	Generalized dystonia and status dystonicus.	Bilateral STN	13 months	Improvement of BFMDRS-M from 58 to 20 (65% reduction) with rapid resolution of status dystonicus.
13	Daida et al. (88)	Autosomal dominant GTP cyclohydrolase 1 deficiency	Parkinsonism and significant motor fluctuations with levodopa	Bilateral STN	NR	Improvement of UPDRS III from 19 to 3 (84% reduction) and of UPDRS IV from 11 to 3 (72% reduction).
14	Tormenti et al. (89)	Tyrosyne hydroxylase deficiency	Generalized dystonia, parkinsonism, and significant motor fluctuations with levodopa	Bilateral STN	14 months	Marked improvement of dystonia and reduction of levodopa dose.
15	Dong et al. (90)	Suspected tyrosine hydroxylase deficiency	Generalized dystonia and significant motor fluctuations with levodopa	Bilateral GPi	6 months	Improvement of BFMDRS-M from 65.5 to 3 (95% reduction) and of BFMDRS-D from 22 to 2 (90% reduction). Also, improvement in motor fluctuations.
16	Beaulieu-Boire et al. (45)	Atypical dopa-responsive dystonia	Generalized dystonia, parkinsonism, and significant motor fluctuations with levodopa	Bilateral GPi	12 months	Improvement of BFMDRS-M off medication from 18 to 10 (44% reduction) and worsening of UPDRS III from 10 to 12 (20% increase)
17	Au et al. (91)	Thiamine pyrophosphokinase deficiency	Generalized dystonia and dyskinesias	Bilateral GPi	12 months	“Subjective improvement of 60% reduction in the hyperkinetic movements.”
18	Mahajan et al. (92)	Thiamine pyrophosphokinase deficiency	Generalized dystonia	Bilateral GPi	NR	“Initial benefit on extension posturing that wore off over the next few years.”
19	Benato et al. (93)	Methylmalonic acidemia	Generalized dystonia and status dystonicus	Bilateral STN	42 months	Resolution of status dystonic without recurrence and progressive improvement of dystonia.
20	Benato et al. (93)	Methylmalonic acidemia	Generalized dystonia and status dystonicus	Bilateral GPi	42 months	Resolution of status dystonic without recurrence and progressive improvement of dystonia.
21	Beaulieu-Boire et al. (45)	Methylmalonic acidemia	Generalized dystonia	Bilateral thalamic	12 months	Response in the BFMDRS-M less than 10% and no change in BFMDRS-D. “Substantial impact on opisthotonic posturing and truncal control.”
22	Chakraborti et al. (94)	Methylmalonic acidemia	Generalized dystonia	Bilateral STN	6 months	Subjective improvement of dystonia and pain not matched by BFMDRS.
23	Payne et al. (95)	Phenylketonuria	Dysmetria, and resting and intention tremor in left upper extremity	Right Vim	30 months	Almost complete resolution of tremor in left upper extremity. Activities of Daily Living self-questionnaire improved from 88 to 35 (60% reduction)
24	Aydin et al. (96)	Homocystinuria	Generalized dystonia	Bilateral GPi	7 months	Improvement of BFMDRS from 60 to 5.5 (91% reduction) and total resolution of masseter and laryngeal dystonia.
25	Saraf et al. (97)	Aicardi-Goutières Syndrome	Generalized dystonia	Bilateral GPi	4 months	Improvement of BFMDRS from 43 to 24 (44% reduction)
26	Kyle et al. (98)	POLR3A leukodystrophy	Parkinsonism and significant motor fluctuations with levodopa	Bilateral GPi	3 months	Improvement of UPDRS from 23 to 18 (22% reduction) with resolution of motor fluctuations.
27	van Karnebeek et al. (99)	X-linked Adrenoleukodystrophy	Generalized dystonia	Bilateral GPi	25 months	Response in the BFMDRS-M and BFMDRS-D less than 10%.

BFMDRS: Burke-Fahn-Marsden Dystonia Rating Scale; BFMDRS-M: Burke-Fahn-Marsden Dystonia Rating Scale motor component; BFMDRS-D: Burke-Fahn-Marsden Dystonia Rating Scale disability component; GPi: Globus Pallidus Internus; NR: Not reported; STN: Subthalamic Nucleus; UPDRS: Unified Parkinson’s Disease rating scale.

## 8. Lesioning techniques

Clinical trials in patients with essential tremor and Parkinson’s disease have shown the benefits of thalamotomy (102, 103). A recent systematic review also found benefits of pallidotomy for dystonia (104). In recent years, there has been a resurgence of interest in lesioning techniques, in part due to the development of new technologies such as magnetic resonance–guided high-intensity focused ultrasound (105). While DBS offers advantages such as reversibility, adjustability of stimulation parameters, and a better safety profile for bilateral procedures, it also comes with the potential risks of infections, skin erosion, and hardware malfunction (8, 79, 104, 106). Additionally, DBS programming is time-consuming, expensive, and requires frequent clinic visits. DBS devices also require periodic surgical procedures for battery replacement (9, 104).

Certain characteristics of patients with IEMs may favor lesioning techniques over DBS. Device infections, skin erosion, and hardware malfunction are more common in children and patients with dystonia (79, 104). Dystonia treatment often requires higher stimulation, leading to faster depletion of batteries (107). Finally, many of these patients have limited mobility and are already having frequent clinic visits with multiple specialties. DBS may add excessively to the burden on patients and their families. Reports of lesioning techniques in IEMs are rare and with short follow-up periods. In patients with PKAN, thalamotomy has been useful for treating dystonia and tremor, while pallidotomy has been effective for dystonia and status dystonicus (70, 108–113). Several cases of successful treatment of tremor in WD with Vim thalamotomy have also been reported (70, 111, 114). Pallidotomy has rarely been used in patients with Batten Disease and GA1 (80, 85).

## 9. Discussion

We conducted a literature review to assess the use of surgical treatments for managing movement disorders in patients with IEMs. Not surprisingly, the available evidence is primarily limited to single cases or case series of selected patients. In most cases, studies have focused on the use of pallidal stimulation for treating dystonia. Overall, GPi-DBS appears to provide benefit for dystonia in PKAN, with sustained benefit several years after the procedure (26, 29). GPi-DBS for LND is also promising, with more notable effects observed in SIB than in dystonia (58). Although there are multiple reports of successful DBS use in other IEM and for other movement disorders, the sample sizes have generally been small, precluding meaningful conclusions. Notably, other surgical interventions, like baclofen pumps, were beyond the scope of this review but may be beneficial in specific patients. Similarly, novel surgical interventions, like neural bypasses, represent intriguing future approaches for patients with movement disorders (115).

Although prevention of neurological dysfunction and pharmacological treatment of movement disorders remain the primary focus for IEMs, the use of DBS should be considered on an individual basis and after open discussions with families of patients with severe refractory movement disorders. Objective measures of movement disorder severity, such as the BFMDRS, should be obtained pre- and post-operatively. However, the extent of DBS benefit may be missed by purely motor scales. Therefore, it is also important to track patient-specific goals, non-motor symptoms, caregiver burden, and QoL. Surgical management should be strongly considered for refractory status dystonicus, which can be life-threatening, as multiple reports have shown symptom resolution in various patients with IEMs (7, 26, 93).

An increased understanding of the effect of surgical techniques for treatment of movement disorders in IEMs would provide additional guidance for providers and families considering these procedures. Given the rarity and heterogeneity of these conditions, clinical trials are unlikely. However, relying solely on case reports and series of selected patients significantly increases the risk of publication bias and may overestimate the effectiveness of DBS. Consistent with this, the first large case series of consecutive PKAN patients showed a lower effect of GPi-DBS than initial reports, although still significant (27). The literature on DBS in LND also suggests that cases with less favorable outcomes are not being reported (58, 59). Encouraging the publication of negative results and large series of consecutive patients is crucial to increase knowledge in this area. This is likely to require multi-institutional collaboration, as it is improbable that a single center will have more than a handful of these patients. Another limitation is that reported follow-up periods are often short. Due to the progressive nature of many of these conditions, long-term outcome reports are needed. Finally, the exploration of the metabolic effects of DBS may be of unique interest in IEMs. DBS can alter regional glucose uptake and neurotransmitter production. A better understanding of these changes could provide valuable guidance for the use of DBS in IEMs that impair energy production or monoamine synthesis (116).

While most of the literature has concentrated on pallidal stimulation for dystonia, it would be valuable to explore further alternative surgical options. First, given the high frequency of basal ganglia injury, the effect of stimulation of other targets such as the STN is an intriguing question. Second, it is important to examine the effect of DBS on other movement disorders, either presenting alone or in combination with dystonia, as many patients with IEMs present with complex movement disorders. Third, the effect of DBS on comorbid neuropsychiatric symptoms should also be studied. Fourth, assessing the utility of new DBS technologies, such as local field potential recording and directional leads, may provide additional treatment options for this population. Finally, it is important to assess the potential indications of lesioning techniques.

## Author contributions

AZ participated in the conception, literature review, and first draft of this manuscript. AG participated in the conception, and review and critique of the manuscript. Both authors have reviewed and approved the final version of the manuscript.

## Conflict of interest

The authors declare that the research was conducted in the absence of any commercial or financial relationships that could be construed as a potential conflict of interest.

## Publisher’s note

All claims expressed in this article are solely those of the authors and do not necessarily represent those of their affiliated organizations, or those of the publisher, the editors and the reviewers. Any product that may be evaluated in this article, or claim that may be made by its manufacturer, is not guaranteed or endorsed by the publisher.
